# Durability and Failure Modes of IPS e.max Endocrown in Endodontically Treated Molars: A 10-Year Clinical Report of Two Cases

**DOI:** 10.7759/cureus.110030

**Published:** 2026-06-01

**Authors:** Rana Essa Dalol, Jihad Nouman Abou Nassar, Mohammad Y. Hajeer

**Affiliations:** 1 Department of Fixed Prosthodontics, Faculty of Dentistry, Syrian Private University, Damascus, SYR; 2 Department of Fixed Prosthodontics, Faculty of Dentistry, University of Damascus, Damascus, SYR; 3 Department of Orthodontics, Faculty of Dentistry, University of Damascus, Damascus, SYR

**Keywords:** clinical success, damaged molar teeth, endocrown, endodontically treated teeth, ips e.max press, restorations

## Abstract

Restoring endodontically treated teeth (ETT) presents a common challenge in general practice. Whereas the traditional method of using dental posts and full-coverage crowns is well-established, it often runs against the minimally invasive principles of recent adhesive dentistry. A more conservative option is provided by endocrowns, which are monolithic restorations that use the pulp chamber for retention.

This study evaluated the long-term clinical performance of two endocrowns (#26, #46) over 10 years. Both restorations were fabricated using the IPS e.max Press system (Ivoclar Vivadent, Schaan, LIE) and cemented with a dual-cure resin cement. Their performance was assessed at baseline, sixth, 12, 18, 60, 84, and 120 months using the modified United States Public Health Service (USPHS) criteria.

At the sixth, 12th, 18th, and 50th-month evaluations, both endocrowns demonstrated 100% clinical success. They showed no issues with marginal adaptation, contact points, surface texture, color match, or retention. However, at the 84-month follow-up, endocrown #26 debonded and required replacement, while the restoration #46 survived until the 120-month follow-up, at which point it failed due to a buccal fracture. In conclusion, these findings indicate that while endocrowns can provide excellent short- to medium-term outcomes, their very long-term survival is challenged by the risks of debonding and fracture, underscoring the importance of meticulous case selection and adherence to adhesive protocols.

## Introduction

There are several methods to restore endodontically treated teeth (ETT) in every clinical scenario. The decision is based on the tooth's protective necessities, appearance, and structural integrity [1]. Endocrowns, direct composite restorations, onlays, overlays, and traditional post and core crowns are some of the restorative options available for severely damaged, endodontically treated molars [1,2]. A full-coverage crown, with or without a post, was the gold standard for restoring (ETT) with significant tissue loss for many years [2]. But since then, principles have changed in favor of more conservative methods due to more emphasis on tooth preservation and improvements in adhesive dentistry [3]. From this viewpoint, the heat-pressed ceramic monoblock procedure was first introduced by Pissis in 1995 [4]. In order to achieve micromechanical retention, the pulp chamber was adhesively bonded to the intact tooth structure, and it was used to increase the macromechanical retention of the crown without intraradicular posts. In 1999, Bindl and Mörmann named the healing process 'endocrown' [5].

The advent of computer-aided design (CAD) and computer-aided manufacturing (CAM), and press systems for ceramics enabled the fabrication of single-unit restorations with high biocompatibility and excellent mechanical properties [6]. Later, glass ceramics became the material of choice for endocrowns, as their surfaces can be effectively modified using hydrofluoric acid etching or air abrasion, thereby enhancing adhesion to tooth tissues. According to the literature, leucite- or lithium disilicate-reinforced glass ceramics have been the best option for fabricating endocrowns. They offer superior flexural strength compared to feldspathic ceramics and resin composites, enabling them to withstand occlusal forces during mastication [7,8].

Restoring non-vital posterior teeth, mainly those with minimum crown height and adequate tissue available for stable, long-term adhesive cementation, the endocrown has been accepted as a viable substitute for full crowns [9]. However, a number of studies [5,10-13] show that these procedures are simpler and faster to conduct than standard single crowns with posts and cores, despite the fact that few specialists feel comfortable performing them. Nevertheless, the absence of high-quality primary research across various materials, heterogeneity within studies, and diversity in follow-up, preparation, and operational stages restricted the literature from developing clear conclusions about the results of endocrowns. The purpose of this case study was to assess the long-term clinical performance of two IPS e.max Press (Ivoclar Vivadent, Schaan, LIE) endocrowns during a 120-month follow-up period in endodontically treated damaged molars.

## Case presentation

Case one

**Figure 1:**
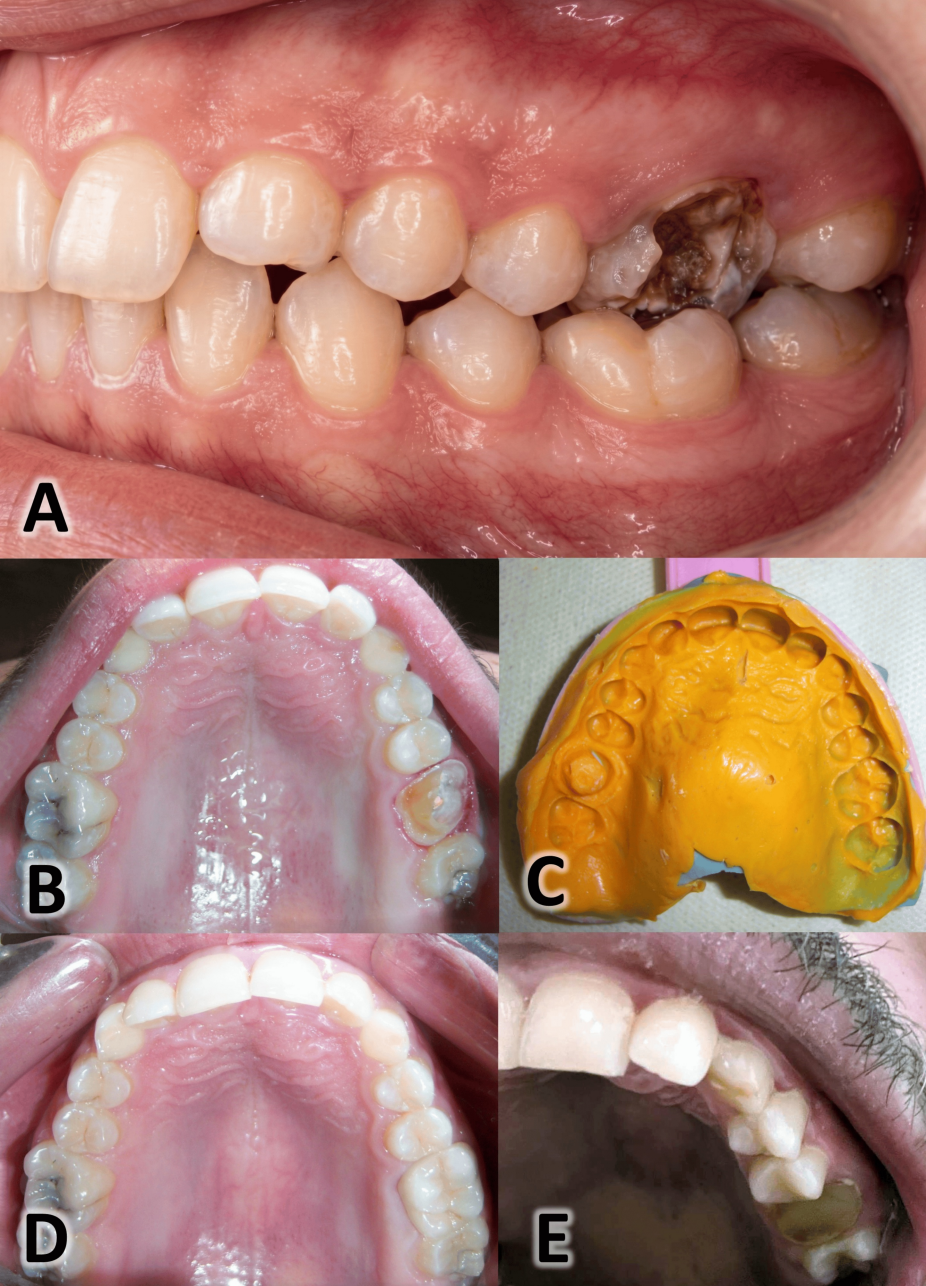
Clinical aspects of an endocrown for the upper left first molar from preparation to placement A: An endodontically treated maxillary first left molar; B: Endocrown preparation; C: The final condensation silicone impression; D: The cemented endocrown; E: Adhesive failure at the seventh-year follow-up

A 20-year-old male in good systemic health presented to the Department of Fixed Prosthodontics, Faculty of Dentistry, University of Damascus (Damascus, SYR), for restoring his compromised maxillary first left molar (tooth #26). Clinical examination revealed that the teeth were asymptomatic and restored with temporary filling materials following completion of endodontic treatment. The remaining coronal tooth structure had an occluso-gingival height of approximately 4.5 mm (Figure 1A).

After removal of the temporary fillings and any unsupported or non-adherent dentin, the pulp chambers were assessed. Their morphology was wide and deep, with dimensions favorable for an adhesive restorative approach. The patient was comprehensively informed of the post-endodontic restorative options, which included a post and core system with a full-coverage crown, as well as more conservative, adhesively retained alternatives such as onlays or endocrowns.

A conservative treatment plan utilizing a lithium disilicate (IPS e.max Press) endocrown was selected. This decision was based on the favorable presentation: the presence of approximately half of the residual coronal structure, the absence of significant occlusal wear facets or parafunctional habits, and pulp chamber anatomy suitable for macro-mechanical retention. The patient's young age and good oral hygiene further supported the long-term prognosis for this adhesive, tooth-preserving approach. The patient was informed about the endocrown restoration using a standardized, detailed information sheet and provided consent, acknowledging that comprehensive long-term clinical success data for this technique remain limited.

Prosthetic decision

Endocrown Preparation

Prior to tooth preparation, the shade was selected using a color guide (VITA Zahnfabrik, Bad Säckingen, DEU), taking into account neighboring and opposite teeth. A lithium disilicate endocrown (IPS e.max Press) was arranged for the upper left first molar (#26) via the following process. The pulp chamber and internal sides of the remaining axial walls were prepared to confirm 6° to 10° of open access with a central retention cavity and rounded internal line angles using a round-end tapered bur #850.010C after 2 mm was removed from the occlusal surface using a fissure bur #835.010 SC (NTI Diamond Instruments, NTI-Kahla GmbH, Kahla, DEU). The same bur was used to cut 1.5 mm from the axial walls' external sides, resulting in a circumferential depth that is a 0.8 mm wide chamfer. After the preparation was complete, the axial walls were polished with a smooth diamond cylinder bur #836kr.314.014 (Komet Dental, Gebr. Brasseler, Lemgo, DEU) (Figure 1B). The interocclusal relationship was captured using bite wax (Tenatex Red; Kemdent, Swindon, Wiltshire, GBR) in the centric occlusion position. Retraction cords were placed (Ultrapake #00, Ultradent, South Jordan, UT, USA), and impressions were made with condensation silicone (putty/light body Zetaplus Oranwash; Zhermack, Badia Polesine, Veneto, ITA) using the two-step technique (Figure 1C).

Bonding Process

Dual-cure resin cement (Variolink; Ivoclar Vivadent) was used to bond the completed endocrown after it had been examined for occlusion, fit, and shade in the patient's mouth. Isolation was achieved using a high-volume suction, cotton rolls in the vestibule and floor of the mouth, and dry angles covering the parotid ducts. The internal surfaces of the endocrowns were treated with the technique indicated for ceramics based on lithium disilicate. After cleaning the extra cement with a disposable applicator, the occlusal, buccal, and lingual surfaces were exposed to light activation for 40 seconds (Figure 1D).

Case two

A 27-year-old healthy male presented to the Restorative Clinic of the Faculty of Dentistry, University of Damascus (Damascus, SYR), for the management of tooth #46. The chief complaint was a two-week history of worsening, sharp pain localized to the tooth, particularly triggered by cold and hot beverages.

Intraoral examination revealed the tooth was acutely sensitive to an air syringe blast but not tender to percussion. Diagnostic testing showed an exaggerated response to electric pulp testing and cold testing (ethyl chloride), with lingering pain persisting for several minutes after stimulus removal. The patient rated the pain intensity as 7 out of 10. A diagnosis of symptomatic irreversible pulpitis with normal apical tissues was established. Following informed consent, non-surgical root canal treatment was initiated.

**Figure 2:**
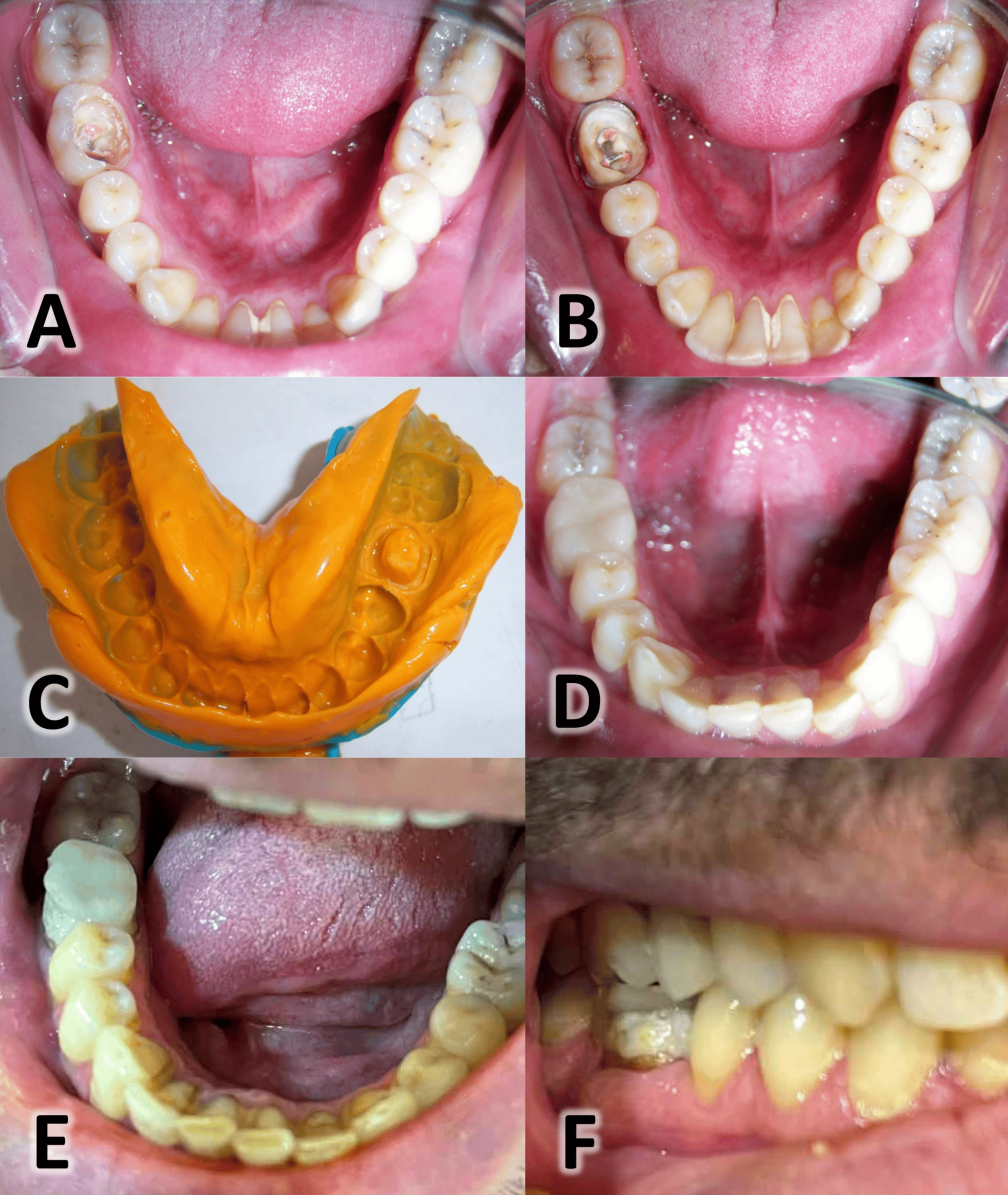
Clinical aspects of an endocrown for the lower right first molar from preparation to placement A: An endodontically treated right mandibular first molar; B: Endocrown preparation; C: The final condensation silicone impression; D: The cemented endocrown; E: Color match failure at the 10th-year follow-up; F: Surface texture failure at the 10th-year follow-up

As in case one, the patient was informed of the post-endodontic restorative options, which included a post and core system with a full-coverage crown versus more conservative, adhesively retained alternatives such as onlays or endocrowns. A conservative treatment plan utilizing an IPS e.max Press endocrown was selected for this molar. This decision was based on favorable conditions, including the presence of more than half of the residual coronal structure; minimal occlusal stress, as evidenced by the absence of steep cuspal anatomy, wear facets, or parafunctional habits; and a pulp chamber morphology conducive to macro-mechanical retention (Figure 2A).

As in the previous case, the patient was informed about the endocrown restoration using a standardized, detailed information sheet and provided consent, acknowledging that comprehensive long-term clinical success data for this technique remain limited. The endocrown preparation (Figure 2B) and final impression (Figure 2C) were performed following the same protocol as detailed in the preceding case. The finished endocrown was checked for shade, fit, and occlusion in the patient's mouth. Following isolation, the endocrown was adhesively cemented using a dual-cure resin cement (Variolink) according to the manufacturer's instructions. Excess cement was meticulously removed with a disposable applicator, and the cement was light-activated for 40 seconds from the occlusal, buccal, and lingual aspects (Figure 2D).

Outcome measures

The endocrowns were assessed using the modified US Public Health Service (USPHS) standards [14-16], which included evaluations of adhesive failure, surface quality, contact points, marginal adaptation, and color match. The following criteria were used to evaluate each parameter. Alfa indicates remarkable clinical performance with no discernible problems; Bravo indicates good clinical performance with minor problems that don't significantly affect the duration or functionality of the restoration; Charlie indicates a restoration with obvious flaws, whereas Delta indicates a treatment failure defined by serious issues that call for rapid repair or restoration replacement.

The endocrowns were assessed at baseline and after six, 12, 18, 60, 84, and 120 months. The clinical examination included radiographs to evaluate the mesial and distal marginal adaptation, a mouth mirror and explorer (EXD56, Hu-Friedy, Chicago, IL, USA), waxed dental floss (Essentialfloss, Oral B, Cincinnati, OH, USA), and a color guide (Vita Lumin vacuum shade guide; Panadent Corp., Colton, CA, USA). Table 1 displays the evaluation of the restorations at recall appointments based on the modified United States Public Health Service (USPHS) standards [14-16].

**Table 1:** Evaluation of endocrowns in recall appointments according to the modified USPHS criteria A: Alfa (clinically excellent restoration); B: Bravo (clinically acceptable restoration); C: Charlie (clinically unacceptable restoration); D: Delta (treatment failure) USPHS: United States Public Health Service

Modified USPHS criteria	Case one (upper left first molar)		Case two (lower right first molar)	
	Baseline	7th year	Baseline	10th year
Color match	A		A	C
Marginal adaptation	A		A	B
Surface texture	A		A	C
Contact points	A		A	B
Adhesive failure	A	D	A	A

Results

Case One (#26)

At the 7th-year recall, the endocrown on tooth #26 was assessed as a clinical failure. While the parameters for color match, marginal adaptation, surface texture, and contact points all received ratings of Alfa at the preceding recall, the assessment for adhesive failure was rated as Delta. This critical finding indicates that the restoration had failed due to loss of retention, requiring replacement. The tooth was subsequently restored with a direct composite core foundation in preparation for a full-coverage crown (Figure 1E).

Case TWO (#46)

At the 10th-year recall, the endocrown on tooth #46, while still functional, exhibited changes that ultimately necessitated its replacement. The assessment of adhesive failure was rated as Alfa, confirming that the restoration remained retained with no signs of debonding after 10 years of clinical service. Marginal adaptation and contact points were rated as Bravo, indicating clinically acceptable discrepancies, including a slightly detectable marginal gap and light proximal contact, with no evidence of secondary caries or periodontal pathology. The color match was rated as Charlie, indicating a clinically unacceptable mismatch in shade (>1 Vita-shade off) (Figure 2E). The surface texture was rated as Charlie due to a fracture on the buccal surface (Figure 2F). This fracture, combined with the unacceptable color match, necessitated the replacement of the restoration.

## Discussion

The well-documented drawbacks of aggressive tooth reduction for full-coverage crowns and the inherent limitations of post and core systems [17,18] have driven the search for more conservative, adhesive alternatives, such as endocrowns and partial restorations. However, the comparative long-term clinical performance of these conservative ceramic restorations remains inadequately established [19], underscoring the need for longitudinal studies.

This case report evaluates the long-term clinical performance of endocrowns as a conservative treatment modality for endodontically treated molars with substantial coronal damage. Conventionally, teeth exhibiting significant loss of two or more dentinal walls are restored with a post and core foundation and a full-coverage crown [20,21]. Endocrowns offer a minimally invasive alternative, preserving residual tooth structure by using the pulp chamber for macromechanical retention.

The two presented cases were selected for endocrown restoration based on established clinical indications. These included molars with cervical margins located at or above the gingival level, enabling the preparation of a well-defined finish line; limited interocclusal space; minimal exposure to functional or lateral stresses; and a pulp chamber depth of at least 3 mm to ensure adequate macro-mechanical retention [21].

Lithium disilicate (IPS e.max Press) was used in this research because it is one of the recommended ceramic materials for manufacturing the endocrown [4]. According to reports, lithium disilicate ceramics belong to the best choices for restoration due to their adhesive qualities [22, 23]. Because it has been demonstrated to imitate pulp chamber details more accurately than the CAD/CAM methods, the pressable ceramic system utilizing the lost-wax technique was used in this study [24,25].

The clinical performance of the endocrowns in this research was assessed using the modified USPHS criteria. These standards allow for the reliable and repeatable evaluation of factors such as marginal adaptation, color match, surface texture, and adhesive integrity. They are well-established and have been actively used in earlier clinical research for the standardized assessment of dental restorations [14-16].

This case report demonstrated a high medium-term survival, with restoration of tooth #26 experiencing complete adhesive failure after 84 months. This result corresponds with Malament et al.'s retrospective study, in which the authors examined and compared the 16.9 years of survival of posterior pressed IPS e.max Press lithium disilicate glass-ceramic complete and partial coverage restorations (n=2392). They reported that most failures (n=17) occurred within 5.6 years and then declined to only five more failures at up to 7.9 years [19]. The concurrence of these failure timelines suggests that the period up to approximately five to seven years represents a critical window for adhesive integrity, beyond which surviving restorations may demonstrate extended longevity.

The adhesive failure observed in this case underscores the multifactorial nature of endocrown success, where material suitability is necessary but not sufficient. While lithium disilicate (IPS e.max Press) was appropriately selected for its documented adhesive properties [21,26], the fabrication technique itself may increase the risk of debonding. The lost-wax press technique was utilized in this study. However, emerging evidence suggests that CAD/CAM fabrication may offer advantages for adhesive integrity, including superior anatomic contour and surface texture that promote better marginal adaptation and physiological occlusion [27,28]. Therefore, the precision of the manufacturing technique, whether press or CAD/CAM, and its ability to produce an optimally fitting restoration are critical variables that interact with material properties to determine long-term clinical outcomes.

Previous clinical investigations have consistently identified debonding as a principal cause of failure [9,29]. This is corroborated by a long-term systematic review by Mario et al., which reported adhesive failure in a significant number of endocrowns after seven to 19 years of service [30]. The biomechanical rationale for this recurring failure is elucidated by finite element analysis. Studies such as the one conducted by Zarone et al. have demonstrated that the restoration-cement-dentin interface is a critical stress-concentration zone in restored ETT, predisposing them to loss of retention [31]. Bonding to the pulp chamber floor and sclerotic dentin, common substrates in these clinical scenarios, presents a significant challenge. Multiple studies have demonstrated that bond strengths in these areas are significantly lower than those of normal coronal dentin, and morphological analyses have revealed suboptimal hybrid layer formation [32-34].

The findings of the present case report directly align with this mechanistic understanding. The observed adhesive failure at the dentin-cement interface strongly implicates the quality and quantity of the remaining dentin substrate as the decisive factor. This underscores that the long-term integrity of suitable ceramic material and the optimal bonding protocol are ultimately governed by the biological foundation to which it adheres.

Isolation is another potential cause of the endocrown debonding. This study aimed to mimic a realistic clinical scenario in a university hospital or specialized prosthodontic office, even though the rubber dam is regarded as the gold standard. In this study, isolation was achieved for all cementation operations using high-volume suction, cotton rolls in the vestibule and on the floor of the mouth, and dry angles positioned over the parotid ducts. The results of this research highlight the important role of using a rubber dam to ensure optimal isolation throughout the bonding process.

A color mismatch was observed in the endocrown (#46) after 120 months between the crown and the adjacent teeth, with a Charlie grade (>1 Vita-shade off) recorded. Several causes were recorded, including changes in the dual-cure resin cement beneath the crowns [35], deterioration of the crowns' external staining layer [36], color changes, and natural teeth's clarity in the oral environment [37], in addition to other personal factors, including smoking, dental health, and food. Therefore, both surface and subsurface causes contribute to color variation. Even though the monolithic IPS e.max Press ceramic exhibits exceptional color stability in vitro, its external characterization stains are at risk of degrading over time [38-40].

The buccal fracture observed in endocrown #46 aligns with established literature identifying fracture as a primary failure mode for these restorations. This finding is corroborated by multiple systematic reviews, which report fractures occurring within follow-up periods ranging from a few years to nearly two decades [12,41,42]. The propensity for fracture may be intrinsically linked to the material properties of lithium disilicate ceramics. Despite their high strength, these materials are inherently brittle and susceptible to fatigue failure under long-term cyclic occlusal loading. Microcracks can initiate at stress-concentration sites, such as sharp internal line angles or the restoration's surface. These microcracks have the potential to spread and combine under dynamic functional loads, ultimately resulting in severe fracture [43-46]. As a result, even for high-strength ceramic restorations, the fracture in this instance is probably due to fatigue over 10 years of clinical service, underscoring the long-term biomechanical issue.

The high medium-to-long-term success rates reported for ceramic endocrowns are consistent with the 10-year survival of the endocrown on tooth #46, which maintained clinically acceptable characteristics until its final buccal fracture. Large-scale retrospective investigations and systematic reviews support this viability; reported survival rates over up to 10 years range from roughly 91% to 98.8% [13,47]. Notably, these positive aggregate statistics conceal a crucial development in material science: more recent data on high-strength lithium disilicate (IPS e.max) consistently show superior survival, exceeding 93% over a decade [19,48], while earlier studies using feldspathic ceramics reported higher failure rates and fractures over extended periods [49].

## Conclusions

This 10-year case report presents two different long-term outcomes for IPS e.max endocrowns in endodontically treated molars. One restoration survived for 10 years before failing due to cohesive ceramic fracture (material failure), while the other failed at seven years because of debonding (adhesive failure). These findings underscore that endocrowns represent a valid, minimally invasive restorative alternative with the potential for excellent longevity; however, their predictability is not absolute. When used within its defined indications, the endocrown maintains a dependable restorative alternative. Long-term success depends on optimizing its inherent conservative advantages while carefully managing the risks of fracture and debonding that are impacted by the patient's biomechanical environment, the restoration design, and the material selection.
